# Effects of Different Temperatures on the Physiological Characteristics of Sweet Potato (*Ipomoea batatas* L. Lam) Storage Roots and Growth of Seedlings During the Sprouting and Seedling Period

**DOI:** 10.3390/plants14060868

**Published:** 2025-03-10

**Authors:** Guangyan Sun, Yi Gou, Linxi Zhang, Mingjun Tang, Yucui Li, Yiming Song, Shuwen Deng, Kang Du, Changwen Lv, Daobin Tang, Jichun Wang

**Affiliations:** 1 College of Agronomy and Biotechnology, Southwest University, Chongqing 400715, China; sunguangyan98@163.com (G.S.); 15523686763@163.com (Y.G.); zhanglinxi2023@163.com (L.Z.); tomjun17@163.com (M.T.); lyc1037654289@163.com (Y.L.); 19139990855@163.com (Y.S.); d86996@email.swu.edu.cn (S.D.); dkk565915482@126.com (K.D.); lvcgwn@swu.edu.cn (C.L.); tdbin741023@163.com (D.T.); 2 Key Laboratory of Biology and Genetic Breeding for Tuber and Root Crops in Chongqing, Chongqing 400715, China

**Keywords:** sweet potato, temperature, sprouting, seedling, starch metabolism

## Abstract

Seedling cultivation is the foremost part of sweet potato (*Ipomoea batatas* L. Lam) production. It is of great significance to reveal the effects of different temperatures on the nutrients of sweet potato storage roots and their relationship with the sprouting quality and to explore the appropriate temperature management for seedlings. In this study, we simulated the temperature differences during the sprouting and seedling period in the summer growing area of sweet potato in the Yangtze River Basin and set three constant temperatures (17 °C, 22 °C and 27 °C) and corresponding three-day/night variable temperatures (21/13 °C, 26/18 °C and 31/23 °C). Thus, we investigated the nutrients, amylase activity, endogenous hormones, and sprouting characteristics of storage roots during the sprouting and seedling period of three sweet potato cultivars with different starch contents. The results showed that with the increase in temperature, the starch and soluble protein (SP) contents in sweet potato storage roots decreased, and the total soluble sugar (TSS), reducing sugar (RS), and sucrose contents increased during the sprouting and seedling period. The amylase activity enhanced; the hormone (IAA) content increased, and the abscisic acid (ABA) content decreased, which, in turn, led to an earlier time of sprouting time (ST), emergence stage (ES), and full stand of seedling stage (FSS). Comparing at the same average temperature, the physiological metabolism and sprouting time and quality of sweet potato were better at variable temperatures than at constant temperatures, in which 31/23 °C was more conducive to the advancement of the ST of sweet potato. At the same time, it was more conducive to the improvement of the seedling cutting amount (SCA), seedling weight (SDW), and seedling number (SDN). The sprouting time and quality of different sweet potato cultivars differed, and cultivars with higher starch content were superior to those with lower starch content. The sucrose and starch contents at different sprouting stages of storage roots can be used as important indicators of the quality of sweet potato seedlings.

## 1. Introduction

Sweet potato (*Ipomoea batatas* L. Lam) is an important crop for food, feed, industrial raw materials, and new energy. It is widely grown in more than 100 countries and regions in the world and is one of the world’s three major tuber and root crops [1]. China is the world’s largest sweet potato producer, with a total sweet potato output of 46.82 million tons in 2022, accounting for about 54.19% of the world’s total output [2]. In sweet potato production, seedling cultivation is the first link and the basis for realizing a high yield of sweet potatoes. It is mainly carried out using storage roots, and then the seedlings are cut and transplanted. The sprouting time and the seedling amount can directly affect the quality and quantity of cutting seedlings. Temperature and cumulative temperature play an important role in controlling the sprouting of underground tuber crops and have an important regulatory effect on the sweet potato dormancy–sprouting cycle [3,4,5]. Generally speaking, the sprouting temperature of sweet potato storage roots should not be lower than 15 °C and higher than 35 °C, and the appropriate day and night temperatures are necessary to promote the rapid and uniform development of the sweet potato root system [6].

The underground storage organs store mainly starch and sugar derivatives, while the seeds store mainly sugars, fats, lipids, and proteins [7]. Underground storage organ sprouting and seed germination/sprouting are similar in many ways, but the main difference is in the utilization of the stored material. Plants finely regulate sugar metabolism to balance dormancy and germination [8,9]. Starch and sugar are key components of tuberous crop storage and are essential for shoot growth. Sprouting (or dormancy) in tuberous plants is associated with the degradation of stored (complex) forms of sugar into mobile and energy-providing (simple) sugar derivatives. Sugar accumulation in storage organs is influenced by external environmental factors, such as temperature, which regulates hormones, sugars, and metabolites in the storage organs, thus affecting germination [7]. Studies have shown that temperature is one of the most important physical factors in determining the length of potato dormancy and that the duration of potato dormancy is inversely proportional to temperature in the range of 3–20 °C [10,11]. High-temperature treatment at 33 °C was more conducive for germination on Konjac Bulbil, and the starch content of the bulb declined at a faster rate in the constant temperature treatment than the diurnal variable temperature treatment [12]. The number of sweet potato seedlings was large under high-temperature conditions of 31–33 °C, while the quality of potato seedlings was better under low and medium temperature conditions of 23–25 °C and 17–19 °C [4].

Previous studies on the effect of temperature on the growth of sweet potatoes are mostly concentrated in the storage period or transplanting period, while there have been few studies on the effect of temperature on the physiological characteristics of sweet potatoes and the change laws of related substances during the sprouting and seedling stages. There has been no systematic comparison of the effects of constant temperature and variable temperature, and the detailed mechanism of how constant temperature and variable temperature interact with sugar and starch metabolism to affect the sprouting and seedlings of sweet potatoes is still not completely clear. Therefore, in this study, we set up a number of variable and constant temperature conditions in an artificial climate incubator to fit the temperature changes in the natural environment in Chongqing and selected three cultivars with large differences in starch content to investigate the sprouting and seedling characteristics of sweet potato storage root under different temperatures. Thus, we investigated the changes in nutrients, amylase activity, and endogenous hormone content in storage roots during the sprouting and seedling period at different temperatures, both constant and variable. It can reveal the physiological mechanism of sweet potato storage root sprouting and seedling, elucidate the correlation between the temperature and the characteristics of sweet potato storage root sprouting and seedling and the change rule of the relevant substances, and clarify the optimal temperature for sweet potato sprouting and seedling. This is an important theoretical and practical reference value for guiding the selection of the seedling time and mode of sweet potato seedlings in the summer potato area of the Yangtze River Basin.

## 2. Results

### 2.1. Changes in Nutrients of Sweet Potato Storage Roots During Sprouting and Seedling Period

**Figure 1:**
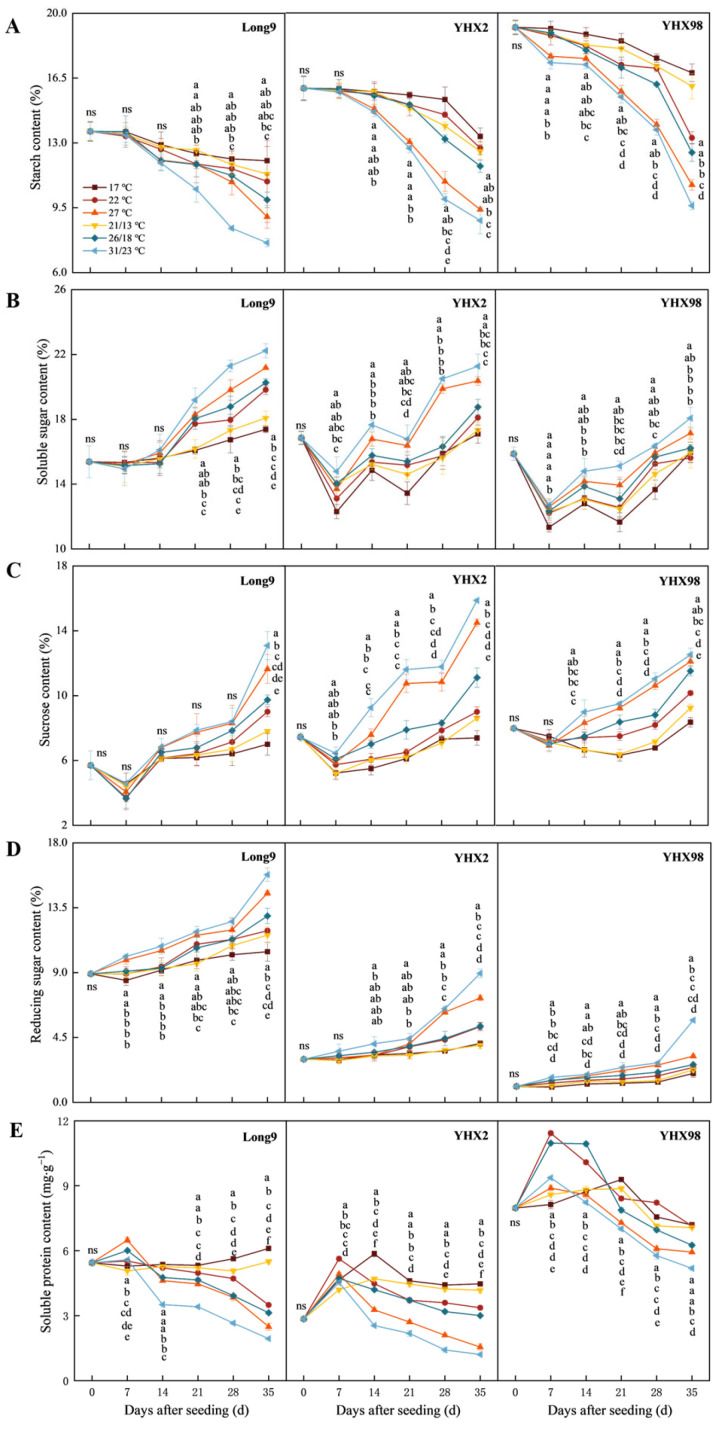
Changes in the content of starch (**A**), total soluble sugar (**B**), sucrose (**C**), and reducing sugar (**D**) soluble protein (**E**) in sweet potato storage roots during sprouting and seedling period. Lowercase letters indicate significant differences in the same cultivar with different temperature treatments with a Tukey test (*p* < 0.05); ns indicates no significant difference.

The starch content of sweet potato storage roots gradually decreased with increasing temperature (Figure 1A). The effects of temperature and cultivar on starch content were highly significant (*p* < 0.01). During the seeding period, the starch content decreased gradually with the increasing seeding time. And at 35 DAS, the decrease rate of starch content of all treatments showed 31/23 °C > 27 °C > 26/18 °C > 22 °C > 21/13 °C > 17 °C, which decreased by 13.59%, 18.20%, 24.35%, 30.01%, 40.28%, and 45.08%, respectively. After 35 DAS, the starch content of the three cultivars showed a decrease in Long9 < YHX2 < YHX98, which was 24.60%, 28.36%, and 31.58%, respectively. And all of them reached the minimum value under the treatment of 31/23 °C, which was 7.61%, 8.81%, and 9.61%, respectively. During the seeding period, the starch content of the three cultivars differed significantly, and all of them showed Long9 < YHX2 < YHX98.

During the seeding period, temperature and cultivar had extremely significant effects on the total soluble sugar (TSS) content of sweet potato storage roots, and the interaction between the two was highly significant on TSS content except for 7 DAS (Figure 1B). TSS content increased with increasing temperature, and the increment of TSS under variable temperature treatment was larger than that under constant temperature treatment at the same average temperature. At 35 DAS, the increase in TSS content of the three cultivars showed Long9 > YHX2 > YHX98, which increased by 28.87%, 10.68%, and 3.90%, respectively. And all three cultivars reached the maximum value at 31/23 °C, which was 22.23%, 21.27%, and 18.07%, respectively.

The sucrose content of sweet potato storage roots decreased and then increased with time during the seeding period in different treatments and reached a minimum at 7 DAS (Figure 1C). The sucrose content increased with increasing temperature. At 35 DAS, the sucrose content of all treatments showed 31/23 °C > 27 °C > 26/18 °C > 22 °C > 21/13 °C > 17 °C. At 35 DAS, the sucrose content of the Long9, YHX2, and YHX98 increased by 70.53%, 42.95%, and 38.85%, respectively, compared with that before seeding, as shown in Long9 > YHX2 > YHX98. And their sucrose content reached the maximum at 31/23 °C, which was 13.09%, 15.87%, and 12.53%, respectively.

The temperature and cultivars had significant effects on reducing sugar (RS) content (Figure 1D). The changing pattern of reducing sugar content in sweet potato storage roots was similar to that of TSS. At 35 DAS, the RS content of all treatments showed that 31/23 °C > 27 °C > 26/18 °C > 22 °C > 21/13 °C > 17 °C. The RS content of Long9, YHX2, and YHX98 all reached the maximum value at 31/23 °C, with 15.79%, 8.95%, and 5.7%, respectively, which were significantly greater than the other treatments. The RS content of the three cultivars before and after seeding differed significantly, with Long9 > YHX2 > YHX98. At 35 DAS, the increase in RS content was Long9 < YHX2 < YHX98, with increases of 43.66%, 93.98%, and 173.83%, respectively.

The soluble protein (SP) content all showed an overall decreasing trend with increasing temperature, and the effects of temperature, cultivars, and the interaction between the two on the SP content of sweet potato storage roots were highly significant (*p* < 0.01) (Figure 1E). During the seeding period, the SP content showed an overall increase and then a decrease trend with the increase in time. The SP content of sweet potato storage roots peaked at 7 DAS and increased compared with that before seeding, except for the 17 °C and 21/13 °C treatments. And the higher the temperature, the greater the decrease, which was 31/23 °C > 27 °C > 26/18 °C > 22 °C, with a decrease of 49.97%, 39.83%, 24.01%, and 14.26%, respectively. Long9, YHX2, and YHX98 all reached the minimum values of 1.94, 1.21, and 4.32 mg·g^−1^ under the 31/23 °C treatment, respectively, which was significantly lower than that of the other treatments. The SP content of different cultivars during the seeding period varied significantly, all of which showed YHX98 > Long9 > YHX2.

### 2.2. Changes in Amylase Activity of Sweet Potato Storage Roots During Sprouting and Seedling Period

**Figure 2:**
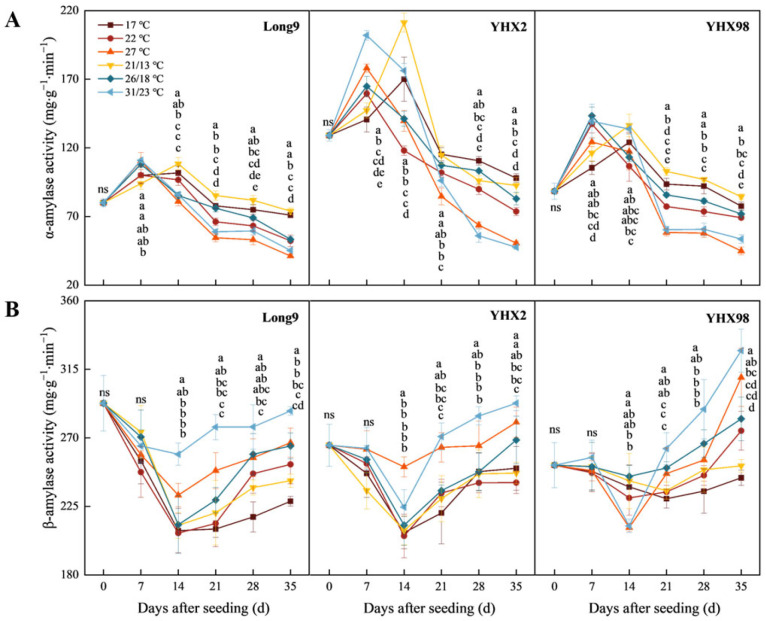
Changes in the activities of α-amylase (**A**) and β-amylase (**B**) in sweet potato storage roots during sprouting and seedling period. Lowercase letters indicate significant differences in the same cultivar with different temperature treatments with a Tukey test (*p* < 0.05); ns indicates no significant difference.

As shown in Figure 2A, the α-amylase activity of sweet potato storage roots was enhanced with increasing temperature in the pre-seeding period and decreased with increasing temperature in the post-seeding period. The effects of temperature, cultivars, and their interaction on α-amylase activity were highly significant (*p* < 0.01). The maximum α-amylase activity of the higher temperature treatments appeared earlier than that of the lower temperature treatments, and the α-amylase activity under the variable temperature treatment was greater than that of the constant temperature treatment at the same average temperature. At 35 DAS, the α-amylase activities of both Long9 and YHX98 reached the minimum at 27 °C, which were 45.26 and 53.63 mg·g^−1^·min^−1^, respectively, and were significantly larger than the rest of the treatments. YHX2 reached its minimum value of 50.63 mg·g^−1^·min^−1^ at 31/23 °C, which was significantly greater than the rest of the treatments, except at 27 °C. Overall, α-amylase activity showed a trend of increasing and then decreasing with the increase in seeding time. The differences in α-amylase activity among the three cultivars during the seeding period were significant, showing that Long9 < YHX98 < YHX2.

β-amylase activity of sweet potato storage roots showed a tendency to decrease and then increase with the increase in seeding time and enhanced with the increasing temperature (Figure 2B). The β-amylase activity was stronger under the variable temperature treatment than the constant temperature treatment at the same average temperature. The β-amylase activity tended to decrease and then increase with the increase in seeding time. Except for YHX98 at 17 °C and 21/13 °C, which reached the minimum at 21 DAS, the other treatments reached the minimum at 14 DAS. The β-amylase activities of Long9, YHX2, and YHX98 were all greater than the rest of the treatments at 31/23 °C at 35 DAS, with the values of 287.67, 292.79, and 327.32 mg·g^−1^·min^−1^, respectively. At 35 DAS, the differences in β-amylase activities of Long9 and YHX2 were not significant, and both were significantly smaller than YHX98.

### 2.3. Changes in Endogenous Hormones of Sweet Potato Storage Roots During Sprouting and Seedling Period

**Figure 3:**
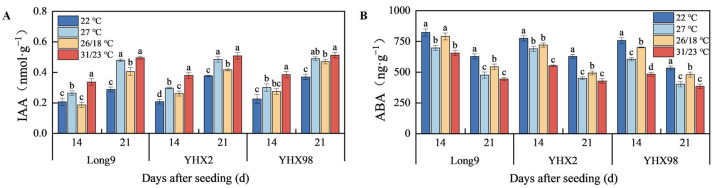
Changes in the contents of IAA (**A**) and ABA (**B**) in sweet potato storage roots during sprouting and seedling period. Lowercase letters indicate significant differences in the same cultivar with different temperature treatments with a Tukey test (*p* < 0.05).

As shown in Figure 3A,B, temperature and cultivar had highly significant effects on IAA and ABA contents of sweet potato storage roots after 14 DAS, and the interaction of the two factors had highly significant effects on IAA contents and significant effects (*p* < 0.05) on ABA contents after 21 DAS. The IAA content of sweet potato storage roots of all treatments showed that 31/23 °C > 27 °C > 26/18 °C > 22 °C. At 21 DAS, the IAA contents of Long9, YHX2, and YHX98 reached the maximum at 23/31 °C, which were 0.49, 0.50, and 0.51 nmol·g^−1^, respectively, and were significantly greater than the rest of the treatments except for 27 °C. The pattern of ABA in sweet potato storage roots was the opposite, with ABA content showing 31/23 °C < 27 °C < 26/18 °C < 22 °C under all treatments. At 21 DAS, the ABA contents of Long9, YHX2, and YHX98 reached the minimum at 31/23 °C, which were 445.09, 428.31, and 385.17 ng·g^−1^, respectively, and were significantly larger than the rest of the treatments except for 27 °C. After 21 DAS, the IAA and ABA contents of the three cultivars differed significantly, with Long9 < YHX2 < YHX98 and Long9 > YHX2 > YHX98, respectively.

### 2.4. Changes in Sprouting Characteristics of Sweet Potato During Sprouting and Seedling Period

The effects of temperature, cultivar, and the interaction of the two on sprouting time (ST), emergence stage (ES), and full stand of seedlings stage (FSS) were highly significant (Table 1). As the temperature increased, the earlier ST, ES, and FSS, as well as the variable temperature treatment, had a stronger effect than the constant temperature treatment for the same average temperature. There were significant differences in ES and FSS among treatments in the three cultivars, all of which showed 22 °C > 26/18 °C > 27 °C > 31/23 °C. However, sweet potato storage roots did not sprout during the seeding period under the 17 °C and 21/13 °C treatments. Under different temperature conditions, the differences in ST, ES, and FSS among cultivars were significant, all of which were Long9 > YHX2 > YHX98.

During the period from 21 to 35 DAS, the temperature had highly significant effects on the hundred seedling weight (HSW) of sweet potato seedlings as well as the seedling cutting amount (SCA) (Table 2). At 35 DAS, all cultivars had seedlings that reached the seedling-cutting standard in all treatments, except for no seedlings in the low-temperature treatment (17 °C and 21/13 °C). Long9 had a significantly larger HSW at 27 °C and 31/23 °C than the rest of the treatments, and YHX2 and YHX98 had a significantly larger HSW at 26/18 °C and 31/23 °C than the rest of the treatments.

As shown in Table 3, the effect of temperature and cultivar on seedling height (SDH), number of expanded leaves (NEL), number of internodes (NI), seedling weight (SDW), and seedling number (SDN) was highly significant. The SDH, NEL, and NI of different cultivars differed significantly under different treatments, all of which were 27 °C > 31/23 °C > 22 °C > 26/18 °C. The SDW and SDN of Long9 and YHX2 all showed 31/23 °C > 27 °C > 26/18 °C > 22 °C under different treatments. The SDW and SDN of YHX98 reached the maximum at 26/18 °C and 31/23 °C, respectively, which was significantly larger than those of the other treatments.

### 2.5. The Relationship Between the Physiological Characteristics and Sprouting Characteristics During Sprouting and Seedling Period

**Figure 4:**
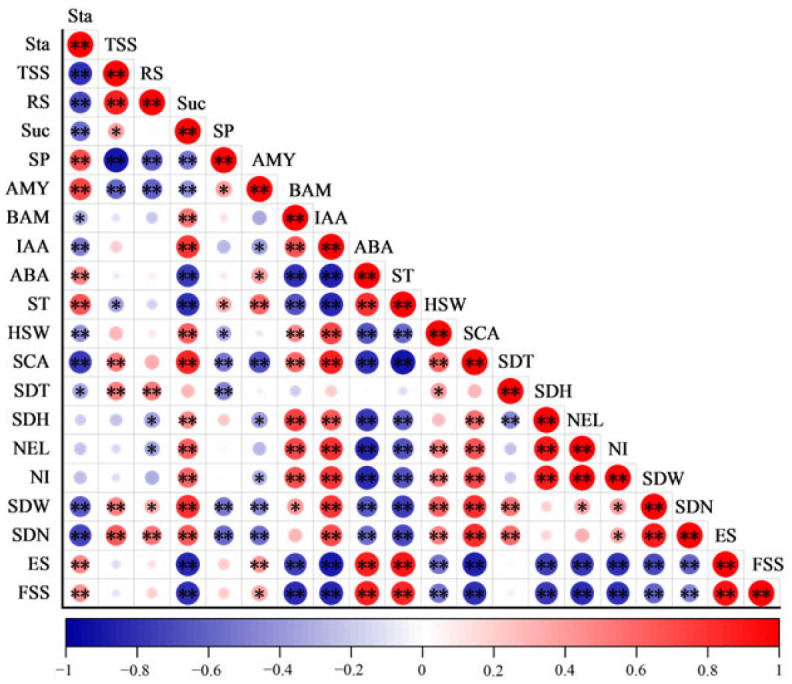
Pearson correlation coefficients between nutrients, amylase activity, hormones, and sprouting characteristics during the sprouting and seedling period. * and ** indicate the significant correlations at the 0.05 and 0.01 levels, respectively. TSS, total soluble sugar; RS, reducing sugar; SP, soluble protein; Suc, sucrose; Sta, starch; AMY, α-amylase; BAM, β-amylase; IAA, growth hormone; ABA, abscisic acid; ST, sprouting time; HSW, 100-seedling weight; SCA, seedling cutting amount; SDT, seedling thick; SDH, seedling height; NEL, number of expanded leaves; NI, number of internodes; SDW, seedling weight; SDN, seedling number; ES, emergence stage; FSS, full stand of seedlings stage.

From Figure 4, it can be seen that the SDW and SDN were negatively correlated with starch, SP, ABA, and ST and positively correlated with SS, sucrose, IAA, HSW, SCA, and SDT. SDW was significantly positively correlated with RS and β-amylase and significantly negatively correlated with α-amylase. The SDN was highly significantly positively correlated with RS and highly significantly negatively correlated with α-amylase.

**Figure 5:**
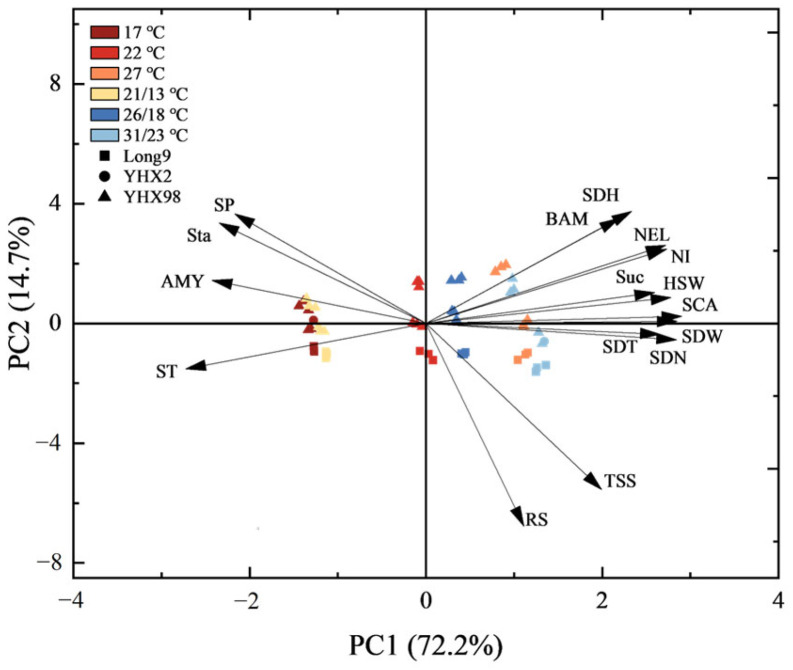
Principal component analysis (PCA) of nutrients, amylase activity, and sprouting characteristics in sweet potato during the sprouting and seedling period. TSS, total soluble sugar; RS, reducing sugar; SP, soluble protein; Suc, sucrose; Sta, starch; AMY, α-amylase; BAM, β-amylase; IAA, growth hormone; ABA, abscisic acid; ST, sprouting time; HSW, 100-seedling weight; SCA, seedling cutting amount; SDT, seedling thick; SDH, seedling height; NEL, number of expanded leaves; NI, number of internodes; SDW, seedling weight; SDN, seedling number. Different colors represent different treatments, and different symbols represent different cultivars.

Principal component analysis (PCA) was used to discriminate the variables related to storage root sprouting in sweet potatoes (Figure 5). The first principal component (PC1) and the second principal component (PC2) retained 72.2% and 14.7% of the variation in data, respectively. SDH, NEL, NI, SCA, SDW, SDT, SDN, TSS, RS, and sucrose showed high positive loadings on PC1, and they were all distributed in the first and second quadrants, indicating a positive correlation with the high-temperature treatment, while the negative loadings of SP, starch, α-amylase activity, and ST on PC1 were high. And they were all distributed in the third and fourth quadrants with the low-temperature treatment, indicating a positive correlation between these four indexes of sweet potato and the low-temperature treatment. Meanwhile, it was observed that high temperature and variable temperatures presented higher positive PC1 scores, while low temperatures presented lower positive PC1 scores, which reflected the role of the high temperature and variable temperatures in promoting sweet potato sprouting. Among the sweet potato cultivars, YHX98 exhibited a higher positive score in PC2, and Long9 had the lowest score.

## 3. Discussion

### 3.1. Effect of Temperature on Starch Metabolism in Sweet Potato Storage Roots

Starch is one of the main components in sweet potato storage roots, and previous studies have shown that α-amylase degraded starch granules into branched chains, maltodextrins, soluble peptides, etc. Subsequently, under the complementary action of β-amylase, starch granules were finally degraded into maltose and low molecular weight dextrins and finally hydrolyzed into glucose by maltogenic enzymes [13,14]. This study showed that with the increase in seeding time, the starch content of sweet potato storage roots gradually decreased; the TSS and sucrose content showed a tendency to decrease and then increase, and the SP showed a tendency to increase and then decrease. This indicates that with the sprouting of storage roots, their internal starch and other nutrients gradually decompose under the action of amylase, thus providing carbon, nitrogen, and energy for the growth of seedlings [15,16,17]. The decrease in TSS content in the pre-seeding stage may be due to the slow starch decomposition rate in this stage within sweet potato storage roots; the utilization of TSS is faster than the starch decomposition of sweet potato; the relative content decreases; the starch decomposition rate in the later stage is faster, and the TSS content gradually increases.

In this study, it was found that temperature affects the decomposition rate of starch and SP in sweet potato storage roots. The higher the temperature, the faster the starch and SP content decreases, and the greater the β-amylase activity, whereas the low temperature delays the degradation of starch [4,18]. Meanwhile, compared with the constant temperature treatment, the starch content of sweet potato storage roots was lower, and the TSS, sucrose, SP contents, and β-amylase activity were higher in the variable temperature treatment. This may be due to the fact that at elevated temperatures as well as at variable temperatures, sweet potato storage roots require more energy to complete their physiological metabolic activities, and the activity of amylase in the storage roots increases, accelerating the decomposition of starch and soluble proteins to small molecules in the sweet potato storage roots, which are run as constituents of the new tissues and raw materials for the energy generation to the growing parts for sprouting and seedling [19]. This also suggests that variable temperature can better regulate the rate of starch degradation in sweet potato storage roots while avoiding low temperature-induced disruption of sugar metabolism.

Previous studies have shown that sugar plays a key role in promoting crop growth [7]. The results of this study were similar; correlation analysis and PCA results showed that the key indicators of seedling quantity and quality had a correlation with the content of sweet potato storage root nutrients, of which starch and sucrose content, followed by RS content, had the greatest influence on seedling quality, which could be used as an additional indicator for detecting the quality of sweet potato seedlings. Among the three cultivars, YHX98 showed the largest decrease in starch content, with the least increase in TSS and sucrose content compared to the other cultivars. This may be because it has a stronger sprouting ability, which requires more sugars in the storage roots to provide energy for sprouting and seedling growth and development.

In addition, the results of this study showed that α-amylase activity was higher in the pre-sprouting period of sweet potato storage roots, and β-amylase activity was higher in the seedling period. And there was a certain positive correlation between the elevation of α- amylase activity and the sprouting of sweet potato storage roots, which played an important role during the sprouting period of sweet potato. This is consistent with the results of Qiu [20].

### 3.2. Effect of Temperature on the Hormones of Sweet Potato Storage Roots

The levels of various endogenous hormones in plants and their relationships with each other are affected by the external environment, including temperature [21,22]. Endogenous hormones are key factors affecting seed viability and germination [23,24]. They respond to multiple physiological changes within the seed by signaling pathways, regulate the metabolism of related proteins and enzymes, and, thus, regulate seed dormancy and germination [25]. Some studies have shown that ABA inhibits the activity of enzymes related to the metabolism of storage material, which limits seed germination and negatively regulates seed vigor [26,27]. IAA promotes the elongation of stems and leaves and the formation of root systems, etc., and the more IAA-containing parts of the plant receive more nutrients and form growth centers [28]. The sprouting of sweet potato storage roots is similarly affected by various endogenous hormones.

In this study, the IAA content of sweet potato storage roots increased during 14–21 DAS, and the IAA content was higher in high temperatures than in medium temperatures. This may be due to the fact that the main function of IAA is to induce plant differentiation of ducts and sieve tubes [29]. As the IAA content of storage roots increased, the rate of transporting nutrients increased, which was conducive to the upward transport of its nutrients by the storage roots to provide nutrients for the growth of seedlings. This can also explain the fact that all three sweet potato cultivars reached the ES and FSS faster during this period under high-temperature conditions than under medium-temperature conditions, suggesting that IAA plays an important role in the sprouting process of sweet potato.

Generally, ABA levels decrease during sprouting in geophytes, and ABA significantly inhibits the α-amylase synthesis transcription and translation period, which affects the sprouting rate of storage roots [7,30]. Studies have shown that the decrease in ABA levels in potato storage roots during sprouting coincided with an increase in TSS [7], which is similar to the results of this study. This study indicated that the decrease in ABA content accumulated in sweet potato storage roots during 14–21 DAS was favorable for the rapid sprouting of sweet potatoes. Meanwhile, under high and variable temperature conditions, the ABA content in sweet potato storage roots was lower than that under medium and constant temperature conditions, and its inhibition of α-amylase activity was weakened, which was more conducive to sweet potato sprouting.

### 3.3. Effect of Temperature on the Sprouting Characteristics of Sweet Potato Storage Roots

Sweet potato sprouting characteristics, as well as seedling quality and quantity, are determined by cultivar characteristics, and temperature also plays a key role. Seeding is burying storage roots in a suitable substrate to raise seedlings; a sprout is the extension of adventitious buds from the root eye through the skin of the sweet potato, and the seedling is the sprouting and growing of buds out of the substrate to a suitable shear for planting. In this study, we found that sweet potatoes under high and variable temperature treatments had the earliest ST, ES, and FSS, which is similar to the results of studies on other crops [19,31,32,33]. In contrast, sweet potato storage roots failed to emerge during the seeding period, although they sprouted normally under the low-temperature treatment. It may be due to the fact that cold damage caused by low temperatures might cause cell membranes to rupture, resulting in the leakage of intracellular water, ions, and metabolites, thereby affecting the sweet potato seedling emergence [34,35]. This indicates that temperature has a significant effect on the sprouting of sweet potato storage roots. Within a certain range, the higher the temperature, the faster the sprouting process of sweet potato storage roots [34]. The results of this study showed that the qualities of sweet potato seedlings under high temperature and variable temperature treatments, such as SDH, NEL, and NI, were significantly greater than those under medium temperature and variable temperature treatments. The results of the PCA also confirmed the roles of high temperature and constant temperature in the promotion of sweet potato sprouting and its quality. Temperature and cultivar were the reasons for the variation in the quality and composition of sweet potato seedlings. In practice, different coverings can be considered to increase the temperature to promote sweet potato sprouting.

The ST of sweet potato cultivars differed significantly only under low-temperature treatments, indicating that the effective accumulated temperature required for sprouting of different sweet potato cultivars differed significantly under low-temperature conditions. The effective accumulated temperature required for sprouting of cultivars with higher amylose content was less than that of cultivars with lower amylose content, whereas the difference was not significant under medium and high-temperature conditions. At the same temperature, the time for different sweet potato cultivars to reach the ES and FSS was YHX98 < YHX2 < Long9, indicating that cultivars with different starch contents had different sprouting and seedling abilities, which were better in cultivars with higher starch contents, which was similar to the results of the studies conducted by Collins et al. [36] and Wang et al. [37]. Under a 27 °C treatment, the SDW and SDT of YHX98 were significantly smaller than those under the other treatments, and the seedlings were susceptible to laxity, slenderness of the stem, elongation of the internodes, and reduction in the seedling quality. This may be due to the fact that constant high temperatures lead to intense respiration and transpiration, which results in the depletion of organic matter, water, and nutrients [38]. Therefore, for cultivars with high starch content, seedlings should be cut in time to ensure that they are strong when they are raised under high-temperature conditions.

## 4. Materials and Methods

### 4.1. Materials

This experiment was conducted in 2022 in the laboratory of the Institute of Tuber and Root Crops of Southwest University. Three fresh sweet potato cultivars with storage root starch contents of about 10%, 15%, and 18%, respectively, were selected for this experiment, namely, Longyan No.9 (Long9), Yuhongxinshu No.2 (YHX2), and Yuhongxinshu No.98 (YHX98). The test materials were planted on 12 May 2022 in Houjiaba, Kaizhou District, Chongqing, and harvested on 14 September 2022, and the storage roots with a uniform shape (average weight of 100–200 g) were selected as seed storage roots. The substrate materials used in this experiment included grass charcoal, vermiculite, and perlite. The organic matter content of grass charcoal was 438.65 g·kg^−1^, and the total nitrogen content was 10.68 g·kg^−1^; the total phosphorus content was 0.36 g·kg^−1^, and the total potassium content was 6.68 g·kg^−1^.

### 4.2. Experimental Design

Grass charcoal, vermiculite, and perlite were mixed and blended in the ratio of 3:1:1 and then packed into a planting box (40 cm × 40 cm × 22 cm, length × width × height), in which 8–10 cm of the substrate was spread flatly. And the seed storage roots were placed flatly in the box and covered with about 5 cm of substrate to keep the moisture content at about 80%, and then they were placed into an artificial climate incubator (RDN-1000B-L2, Ningbo Yanghui Instrument Co., Ltd., Zhejiang, China). Each pot had a small hole at the bottom to allow for the drainage of excess water and nutrients.

This experiment was designed as a two-factor split-zone experiment with six temperature levels as the main factor to simulate the temperature differences during the seedling period in the summer growing area of sweet potato in the Yangtze River Basin. Temperatures were the main factors, and three constant temperatures at 17 °C (low), 22 °C (medium), and 27 °C (high) and three day/night variable temperatures at 21/13 °C (low), 26/13 °C (medium) and 31/23 °C (high) were set up. The subfactors were three cultivars (Long9, YHX2, and YHX98). The day/night duration of the artificial climatic incubator was set at 12 h/12 h; the relative humidity of air was 85%, and the illumination was 10,000 lx with mixtures of green [G (500–600 nm)], blue [B (400–500 nm)], and red [R (600–700 nm)] radiation. Each treatment was replicated three times, and three boxes were used for each replication. The samples were taken after 0, 7, 14, 21, 28, and 35 days after seeding (DAS) to measure the indicators, and the seedlings were uniformly harvested at 40 DAS to record the data.

### 4.3. Measurement Indicators and Methods

#### 4.3.1. Determination of Starch, Total Soluble Sugar, Sucrose, Reducing Sugar and Soluble Protein in Storage Roots

Starch, total soluble sugar (TSS), and sucrose contents were determined by anthrone colorimetry [39]. Reducing sugar (RS) content was determined by 3,5-dinitrosalicylic acid (DNS) colorimetric method [40]. A 0.1 g dry sample was weighed, ground, and put into a 10 mL centrifuge tube. A total of 4 mL of 80% ethanol was added and extracted in a water bath at 80 °C for 40 min, shaking several times during the process. The tubes were centrifuged at 4000 r·min^−1^ for 5 min, and 4 mL of 80% ethanol was added to the precipitate. The previous step was repeated. The supernatants from the two centrifugations were combined and used as an extract solution of TSS, RS, and sucrose, and the obtained residues were used to determine the starch. 

Soluble protein (SP) was determined by Coomassie brilliant blue colorimetry with slight modifications [41]. An amount of 0.5 g of fresh sweet potato storage roots was put in a mortar, ground into a homogenate with 2 mL of distilled water, and transferred to a 15 mL centrifuge tube. Then, the mortar was washed with 6 mL of distilled water several times, and it was adjusted to 10 mL with distilled water. Then, it was centrifuged at 4000 r·min^−1^ for 10 min, and the supernatant was adjusted to 100 mL with distilled water. An amount of 1 mL of supernatant was taken in a 10 mL test tube, adding 5 mL of Coomassie brilliant blue, which was shaken well and stood for 2 min, and then it was measured from the absorbance at 595 nm (UV-1801; Beifen-Ruili, Beijing, China).

#### 4.3.2. Determination of Amylase Activity in Storage Roots

Amylase activity was determined by the colorimetric method with DNS [41,42]. The samples of 0.1 g were ground and homogenized with 1.5 mL of distilled water and then centrifuged for 20 min (20,000× *g*). The supernatant was collected as an enzyme extract for amylase activity determination. The enzyme extract (1 mL) kept at 70 °C for 15 min was mixed with citric acid buffer (1 mL, pH 5.6) and maintained at 40 °C for 15 min. The reaction started when the mixture was mixed with 2 mL starch solution and 1 mL DNS in a boiling water bath for 5 min. The absorbance at 540 nm was measured. The α-amylase activity was calculated by the standard curve.

The determination of total amylase and β-amylase activity was similar to the α-amylase. The reaction system consisted of 1 mL enzyme extract, 2 mL starch solution, and 1 mL DNS, which was kept in a water bath for 5 min. The absorbance value was measured as above to obtain the total amylase. The β-amylase activity was calculated as the following equation: β-amylase activity = total amylase activity − α-amylase activity. One unit of amylase activity was defined as the amount of enzyme required to catalyze the production of 1 g of maltose per minute per gram of tissue at 40 °C.

#### 4.3.3. Endogenous Hormone Measurement

The content of growth hormone (IAA) and abscisic acid (ABA) was determined using the IAA and ABA ELISA Kit (Shanghai Youxuan Biotechnology Co., Ltd., Shanghai, China) with reference to the enzyme-linked immunosorbent assay (ELISA) of He et al. [43]. The sample of 0.5 g was quick-frozen in liquid nitrogen, homogenized with an 80% methanol solution (containing di-tert-butyl-p-cresol (BHT) 1 mmol·L^−1^), extracted at 4 °C for 4 h, centrifuged at 4000 r·min^−1^ for 15 min, and extracted with 80% methanol three times. Then, the supernatants were combined and blown dry under nitrogen gas and dissolved and fixed with phosphate buffer at pH 7.5 (500 mL phosphate buffer with 0.5 mL Tween-20 and 0.5 g gelatin) for ELISA.

#### 4.3.4. Measurement of Sprouting and Seedling Characteristics

The number of days when sweet potato storage roots sprouted was recorded as sprouting time (ST), the number of days when 10% of seedlings emerged was recorded as emergence stage (ES), and the number of days when 70% of seedlings emerged was recorded as full stand of seedlings stage (FSS) [44]. Seedling thickness (SDT) was measured on the stem thickness of the upper, middle, and lower parts of the seedling with a vernier caliper and taking the average value. Seedling height (SDH) was measured on the vertical distance from the base of the seedling to the top growing point with a tape measure in cm. The seedlings on the sampled sweet potato storage roots were removed, washed, and dried, and the storage roots and seedlings above 20 cm were weighed or counted separately. The seedling cutting amount (SCA) was counted on the number of seedlings per storage root above 20 cm. The seedling weight (SDW) = seedling weight per storage root/single storage root weight (g·kg^−1^), seedlings number (SDN) = SCA/single storage root weight (plant·kg^−1^), 100-seedling weight (HSW) = seedling weight per storage root/SCA × 100 (g·100-plants^−1^). The number of expanded leaves (NEL) and the number of internodes (NI) were counted per seedling.

### 4.4. Data Analysis

Statistical analysis was performed by a one-way and two-way ANOVA using SPSS 26.0 (IBM Corp., Chicago, IL, USA). A one-way analysis of variance was conducted to test the effect of sprouting temperature on each measured parameter. The interaction effect of sprouting temperature and cultivars was analyzed by two-way analysis of variance. All data were expressed as mean ± standard deviation (*n* = 3), and the significance of differences was tested using Tukey’s test, taking *p* < 0.05 as significant. Pearson’s correlation test was calculated to analyze the correlation between growth parameters and nutritional quality. Principal component analysis (PCA) was performed to evaluate the effect of the temperature treatment on sweet potatoes. The correlation test and PCA were completed using Origin 2024 (OriginLab, Hampton, MA, USA), and all images were created by Origin 2024.

## 5. Conclusions

In this study, we investigated the effects of different temperatures on the physiological characteristics and growth characteristics of sweet potato seedlings during the sprouting and seedling period. It was found that with the increase in temperature, the amylase activity of sweet potato storage roots was enhanced, the IAA content increased, the ABA content decreased, the decomposition rate of starch and SP accelerated, and the accumulation of carbohydrates increased, resulting in advancement of the ST, ES, and FSS of sweet potato storage roots. The physiological metabolic activity of sweet potatoes under variable temperature treatment was more active than that under constant temperature treatment, which could promote the early sprouting of sweet potato seedlings and improve the quality and quantity of seedlings. The sprouting time and quality of different sweet potato cultivars varied, and cultivars with higher starch content were better than those with lower starch content. There was a certain relationship between the number and quality of seedlings and the nutrient content of sweet potato storage roots, of which the starch and sucrose content had the greatest influence on the quality of seedlings. In seedling production, to obtain robust seedlings, the average temperature of the nursery environment should be maintained at a constant or variable temperature of 22 or 27 °C, and the seedlings should be cut at 35 DAS and 28 DAS, respectively.

## Figures and Tables

**Table 1:** Changes in sprouting time of sweet potato during the sprouting and seedling period.

Cultivars	Temperature	Sprouting Time	Emergence Stage	Full Stand of Seedling Stage
		(d)	(d)	(d)
Long9	17 °C	21.33 ± 1.21 a	n.d.	n.d.
	22 °C	7.33 ± 0.82 c	22.50 ± 1.22 a	27.33 ± 1.21 a
	27 °C	4.50 ± 0.55 d	13.33 ± 1.03 c	20.83 ± 1.33 c
	21/13 °C	19.83 ± 1.17 b	n.d.	n.d.
	26/18 °C	6.50 ± 0.84 c	20.17 ± 1.60 b	25.33 ± 1.21 b
	31/23 °C	4.00 ± 0.63 d	11.33 ± 0.52 d	18.50 ± 0.84 d
YHX2	17 °C	18.33 ± 0.82 a	n.d.	n.d.
	22 °C	6.67 ± 0.82 c	18.00 ± 1.10 a	25.00 ± 0.89 a
	27 °C	4.50 ± 0.55 d	10.50 ± 0.84 c	15.67 ± 1.03 c
	21/13 °C	16.50 ± 1.52 b	n.d.	n.d.
	26/18 °C	6.33 ± 1.03 c	16.50 ± 0.84 b	21.33 ± 1.21 b
	31/23 °C	3.83 ± 0.75 d	8.67 ± 0.82 d	13.00 ± 0.89 d
YHX98	17 °C	16.33 ± 0.82 a	n.d.	n.d.
	22 °C	6.50 ± 0.55 c	16.33 ± 1.21 a	23.50 ± 1.52 a
	27 °C	4.17 ± 0.75 d	9.67 ± 0.52 c	14.17 ± 0.75 c
	21/13 °C	14.17 ± 1.17 b	n.d.	n.d.
	26/18 °C	6.00 ± 1.10 c	15.00 ± 0.63 b	20.50 ± 1.64 b
	31/23 °C	3.67 ± 0.52 d	8.50 ± 0.55 d	11.50 ± 1.05 d
Significance				
Cultivar		**	**	**
Temperature		**	**	**
Cutivar × Temperature		**	*	*

Note: Lowercase letters indicate significant differences in the same cultivar with different temperature treatments with a Tukey test (*p* < 0.05). Data are expressed as the mean ± SE. n.d. indicates no data. The statistical results of two-way ANOVA of variance are expressed in *p*-value. * *p* < 0.05; ** *p* < 0.01.

**Table 2:** Changes in 100-seedling weight and seedling cuttings of sweet potato during the sprouting and seedling period.

Cultivars	Temperature	Days After Seeding					
		21 d		28 d		35 d	
		A 100-Seedling Weight	Seedling Cutting Amount	A 100-Seedling Weight	Seedling Cutting Amount	A 100-Seedling Weight	Seedling Cutting Amount
		(g·100-Plants^−1^)	(Plant)	(g·100-Plants^−1^)	(Plant)	(g·100-Plants^−1^)	(Plant)
Long9	17 °C	n.d.	n.d.	n.d.	n.d.	n.d.	n.d.
	22 °C	n.d.	n.d.	n.d.	n.d.	752.33 ± 23.46 c	2.43 ± 0.09 d
	27 °C	159.67 ± 11.24 b	0.77 ± 0.20 b	591.90 ± 3.86 b	3.50 ± 0.17 b	1177.65 ± 174.54 a	6.35 ± 0.14 b
	21/13 °C	n.d.	n.d.	n.d.	n.d.	n.d.	n.d.
	26/18 °C	n.d.	n.d.	223.27 ± 7.82 c	0.90 ± 0.09 c	1049.98 ± 155.87 b	3.30 ± 0.04 c
	31/23 °C	250.58 ± 10.93 a	1.3 ± 0.05 a	626.33 ± 27.43 a	4.11 ± 0.51 a	1228.86 ± 184.79 a	6.73 ± 0.12 a
YHX2	17 °C	n.d.	n.d.	n.d.	n.d.	n.d.	n.d.
	22 °C	n.d.	n.d.	n.d.	n.d.	762.22 ± 42.34 c	2.47 ± 0.21 c
	27 °C	503.70 ± 1.79 b	1.18 ± 0.17 a	597.13 ± 11.80 b	4.50 ± 0.17 b	1174.23 ± 41.51 b	6.50 ± 0.17 a
	21/13 °C	n.d.	n.d.	n.d.	n.d.	n.d.	n.d.
	26/18 °C	n.d.	n.d.	281.31 ± 5.69 c	1.32 ± 0.21 c	1301.62 ± 49.71 a	3.55 ± 0.19 b
	31/23 °C	568.08 ± 4.63 a	1.33 ± 0.33 a	690.63 ± 21.85 a	5.50 ± 0.17 a	1268.35 ± 35.65 a	6.51 ± 0.17 a
YHX98	17 °C	n.d.	n.d.	n.d.	n.d.	n.d.	n.d.
	22 °C	n.d.	n.d.	320.5 ± 14.91 d	1.43 ± 0.17 d	737.88 ± 57.82 c	2.52 ± 0.17 d
	27 °C	525.42 ± 28.22 b	1.15 ± 0.16 b	832.32 ± 9.14 b	4.66 ± 0.34 b	987.27 ± 97.11 b	5.55 ± 0.09 b
	21/13 °C	n.d.	n.d.	n.d.	n.d.	n.d.	n.d.
	26/18 °C	n.d.	n.d.	452.50 ± 5.68 c	1.77 ± 0.20 c	1327.88 ± 20.27 a	3.72 ± 0.26 c
	31/23 °C	572.72 ± 10.33 a	1.77 ± 0.20 a	999.42 ± 13.49 a	5.50 ± 0.17 a	1264.82 ± 62.69 a	6.19 ± 0.17 a
Significance							
Cultivar		**	*	**	**	ns	*
Temperature		**	**	**	**	**	**
Cultivar × Temperature		ns	ns	**	*	*	**

Note: Lowercase letters indicate significant differences in the same cultivar with different temperature treatments with a Tukey test (*p* < 0.05). Data are expressed as the mean ± SE. n.d. indicates no data. The statistical results of two-way ANOVA of variance are expressed in *p*-value. * *p* < 0.05; ** *p* < 0.01; ns = no significant difference.

**Table 3:** Changes in seedling characteristics of sweet potato during the sprouting and seedling period.

Cultivars	Temperature	Seedling Thickness	Seedling Height	Number of Expanded Leaves	Number of Internodes	Seedling Weight	Seedling Number
		(cm)	(cm)	(Piece)	(Internode)	(g·kg^−1^)	(Plant·kg^−1^)
Long9	17 °C	n.d.	n.d.	n.d.	n.d.	n.d.	n.d.
	22 °C	0.29 ± 0.01 c	19.54 ± 0.99 d	4.66 ± 0.85 d	6.69 ± 0.55 d	197.18 ± 11.52 d	16.7815 ± 0.47 d
	27 °C	0.38 ± 0.02 b	55.97 ± 1.04 a	10.19 ± 0.33 a	15.23 ± 0.43 a	422.38 ± 6.64 b	50 ± 1.44 b
	21/13 °C	n.d.	n.d.	n.d.	n.d.	n.d.	n.d.
	26/18 °C	0.31 ± 0.01 c	29.26 ± 1.6 c	7.41 ± 0.47 c	9.83 ± 0.76 c	289.72 ± 3.13 c	30.8524 ± 0.29 c
	31/23 °C	0.42 ± 0.09 a	39.73 ± 3.33 b	8.68 ± 0.12 b	12.10 ± 0.14 b	489.70 ± 3.43 a	52.076 ± 1.23 a
YHX2	17 °C	n.d.	n.d.	n.d.	n.d.	n.d.	n.d.
	22 °C	0.31 ± 0.02 c	25.27 ± 0.73 d	6.12 ± 0.29 c	8.61 ± 0.38 d	251.09 ± 2 d	26.7118 ± 1.11 c
	27 °C	0.33 ± 0.01 b	73.34 ± 1.02 a	12.71 ± 0.44 a	17.78 ± 0.16 a	355.86 ± 2.27 b	35.21 ± 0.88 b
	21/13 °C	n.d.	n.d.	n.d.	n.d.	n.d.	n.d.
	26/18 °C	0.32 ± 0.02 bc	35.62 ± 2.7 c	9.37 ± 0.23 b	12.58 ± 0.53 c	284.11 ± 4.29 c	28.3169 ± 1.43 c
	31/23 °C	0.35 ± 0.03 a	54.23 ± 0.63 b	9.89 ± 0.62 b	13.36 ± 0.07 b	538.60 ± 11.85 a	45.06 ± 2.20 a
YHX98	17 °C	n.d.	n.d.	n.d.	n.d.	n.d.	n.d.
	22 °C	0.3 ± 0.01 b	40.96 ± 2.41 d	7.77 ± 0.8 d	10.83 ± 0.52 d	279.56 ± 3.94 d	26.84 ± 1.20 c
	27 °C	0.19 ± 0.01 c	131.18 ± 2.2 a	14.64 ± 0.12 a	20.01 ± 0.91 a	308.87 ± 4.89 c	31.03 ± 1.35 b
	21/13 °C	n.d.	n.d.	n.d.	n.d.	n.d.	n.d.
	26/18 °C	0.34 ± 0.01 a	52.78 ± 2.73 c	10.97 ± 0.27 c	14.92 ± 0.72 c	371.95 ± 10.43 a	27.21 ± 0.15 c
	31/23 °C	0.29 ± 0.03 b	95.04 ± 4.68 b	12.24 ± 0.28 b	16.71 ± 0.86 b	353.11 ± 1.94 b	33.66 ± 0.79 a
Significance							
Cultivar		**	**	**	**	**	**
Temperature		*	**	**	**	**	**
Cultivar × Temperature		**	**	ns	ns	**	**

Note: Lowercase letters indicate significant differences in the same cultivar with different temperature treatments with a Tukey test (*p* < 0.05). Data are expressed as the mean ± SE. n.d. indicates no data. The statistical results of two-way ANOVA of variance are expressed in *p*-value. * *p* < 0.05; ** *p* < 0.01; ns = no significant difference.

## Data Availability

Data are contained within this article.

## Abbreviations

The following abbreviations are used in this manuscript:

TSS	Total soluble sugar
RS	Reducing sugar
SP	Soluble protein
Suc	Sucrose
Sta	Starch
AMY	α-amylase
BAM	β-amylase
IAA	Growth hormone
ABA	Abscisic acid
ST	Sprouting time
ES	Emergence stage
FSS	Full stand of seedlings stage
HSW	100-seedling weight
SCA	Seedling cutting amount
SDT	Seedling thick
SDH	Seedling height
NEL	Number of expanded leaves
NI	Number of internodes
SDW	Seedling weight
SDN	Seedling number
